# Development and emerging trends of drug resistance mutations in HIV: a bibliometric analysis based on CiteSpace

**DOI:** 10.3389/fmicb.2024.1374582

**Published:** 2024-05-15

**Authors:** Xuannan Chen, Xi Chen, Yu Lai

**Affiliations:** ^1^Acupunture and Tuina School, Chengdu University of Traditional Chinese Medicine, Chengdu, China; ^2^School of Basic Medicine, Chengdu University of Traditional Chinese Medicine, Chengdu, China

**Keywords:** HIV, AIDS, drug resistance mutation, CiteSpace, bibliometric analysis, visualized analysis, hot research topics

## Abstract

**Background:**

Antiretroviral therapy has led to AIDS being a chronic disease. Nevertheless, the presence of constantly emerging drug resistance mutations poses a challenge to clinical treatment. A systematic analysis to summarize the advancements and uncharted territory of drug resistance mutations is urgently needed and may provide new clues for solving this problem.

**Methods:**

We gathered 3,694 publications on drug resistance mutations from the Web of Science Core Collection with CiteSpace software and performed an analysis to visualize the results and predict future new directions and emerging trends. Betweenness centrality, count, and burst value were taken as standards.

**Results:**

The number of papers on HIV medication resistance mutations during the last 10 years shows a wave-like trend. In terms of nation, organization, and author, the United States (1449), University of London (193), and Mark A. Wainberg (66) are the most significant contributors. The most frequently cited article is “Drug resistance mutations for surveillance of transmitted HIV-1 drug-resistance: 2009 update.” Hot topics in this field include “next-generation sequencing,” “tenofovir alafenamide,” “children,” “regimens,” “accumulation,” “dolutegravir,” “rilpivirine,” “sex,” “pretreatment drug resistance,” and “open label.” Research on drug resistance in teenagers, novel mutation detection techniques, and drug development is ongoing, and numerous publications have indicated the presence of mutations related to current medications. Therefore, testing must be performed regularly for patients who have used medications for a long period. Additionally, by choosing medications with a longer half-life, patients can take fewer doses of their prescription, increasing patient compliance.

**Conclusion:**

This study involved a bibliometric visualization analysis of the literature on drug resistance mutations, providing insight into the field’s evolution and emerging patterns and offering academics a resource to better understand HIV drug resistance mutations and contribute to the field’s advancement.

## 1 Introduction

Human immunodeficiency virus type 1 (HIV-1) is one of the most well-known and infectious causative agents. This virus increases susceptibility to various illnesses and has the potential to cause several cancers by infecting CD4+ cells, depleting them, and weakening the immune system. The equivalent of 40.4 million people have died from HIV thus far, and it is still spreading throughout the world. It was estimated that 39.0 million people were HIV positive as of the end of 2022. There were 630 thousand HIV-related deaths in 2022, and 1.3 million [1.0–1.7 million] people contracted the virus (WHO, 2023). Given the tremendous pace of mutation and the unique infection processes of HIV, there is currently no viable vaccine available. However, allogeneic hematopoietic stem cell transplantation from donors with a homozygous CCR5 delta32 mutation is capable of functionally curing HIV infection (Ding et al., 2021). Due to the high risk and significant financial expenditures of the surgery, widespread clinical use is not realistic (Gavegnano et al., 2019; Gupta and Saxena, 2021).

Fortunately, combination antiretroviral therapy (cART), which was introduced in 1996, has substantially contributed to a decrease in mortality, successfully slowed viral proliferation, lengthened the life expectancy of HIV-positive patients, and ultimately led to HIV infection becoming a controllable chronic illness. Typically, cART inhibits deterioration of HIV by preventing specific critical steps in the virus’s life cycle with multiple antiretroviral drugs, including protease inhibitors (PIs), integrase strand-transfer inhibitors (INSTIs), entry inhibitors, nucleoside reverse transcriptase inhibitors (NRTIs), and nonnucleoside reverse transcriptase inhibitors (NNRTIs) (De Clercq and Li, 2016). However, drug failure can result from insertion mutations, replacement mutations, high mutation rate-induced sequence variation in the capsid protein, and drug-induced modifications to HIV enzymes (Tang and Shafer, 2012; Saito and Yamashita, 2021; Richetta et al., 2022; Dakshinamoorthy et al., 2023). As mutations accumulate, some viruses become resistant, eventually creating new subtypes. This phenomenon is generally referred to as drug resistance mutations (DRMs). It has an impact on clinical applications and raises concerns about current therapies, including the following: (i) most resistance mutations reduce sensitivity to similar-class antiretrovirals, resulting in long-term ART narrowing (Tang and Shafer, 2012); (ii) existing antiretroviral medications become ineffective due to the increase in multidrug resistance, which might further fail drug therapy (Puertas et al., 2020); and (iii) there is a latent prevalence of DRM virus subtypes, delaying the 2030 target date for ending the HIV epidemic globally. Understanding the mechanisms of drug resistance is essential for solving these problems. In general, gene recombination caused by infection of the same cell by parental strains of different subtypes (Nastri et al., 2023) and the high rate of mutations caused by HIV replication in host cells are two factors that contribute to medication resistance. In the past few decades, many studies and techniques have been conducted on the mechanism of HIV resistance mutations. To understand the dynamic evolution of HIV drug resistance mutations and to move the field forward, a systematic evaluation and overview of these studies and approaches are necessary. Thus, a quantitative characterization of the current research landscape, hot research topics, and frontiers of drug resistance mutations is needed.

Numerous areas, including public health and clinical practice, have embraced bibliometrics to examine and evaluate the progress and frontiers of their respective fields. Knowledge domain visualization (KDViz) aims to detect and follow the evolution of the knowledge domain and to identify major changes in the domain easily via bibliometrics. KDViz relies on visual processing technology, such as CiteSpace, which is based on a Java program developed by Professor Chaomei Chen in 2004 (Chen, 2004). Through this program, we can thoroughly evaluate and characterize the research developments and trends among studies and methodologies of DRM, deconstruct the complexity of DRMs, identify critical areas that require attention, and create the foundation for further investigations and issues. Nevertheless, researchers have yet to perform similar bibliometric research, such as CiteSpace-based research on drug-resistant mutations, at least not using available data. Therefore, a thorough discussion of the accomplishments in this area is needed.

## 2 Methods

### 2.1 Data collection

The Web of Science Core Collection (WOSCC) advanced search feature was utilized to find relevant articles on “drug resistance mutations,” as it is the only database fully utilizing CiteSpace’s functionalities (Chen, 2006). The following search phrases were used:

(((((TS = (HIV)) OR TS = (“Human Immunodeficiency Viru* “)) OR TS = (HTLV-III)) OR TS = (“AIDS Viru* “)) AND TS = (“Drug Resistan* “)) AND TS = (Mutation*).

As inclusion criteria, only articles and review articles published in English were included.

**Figure 1:**
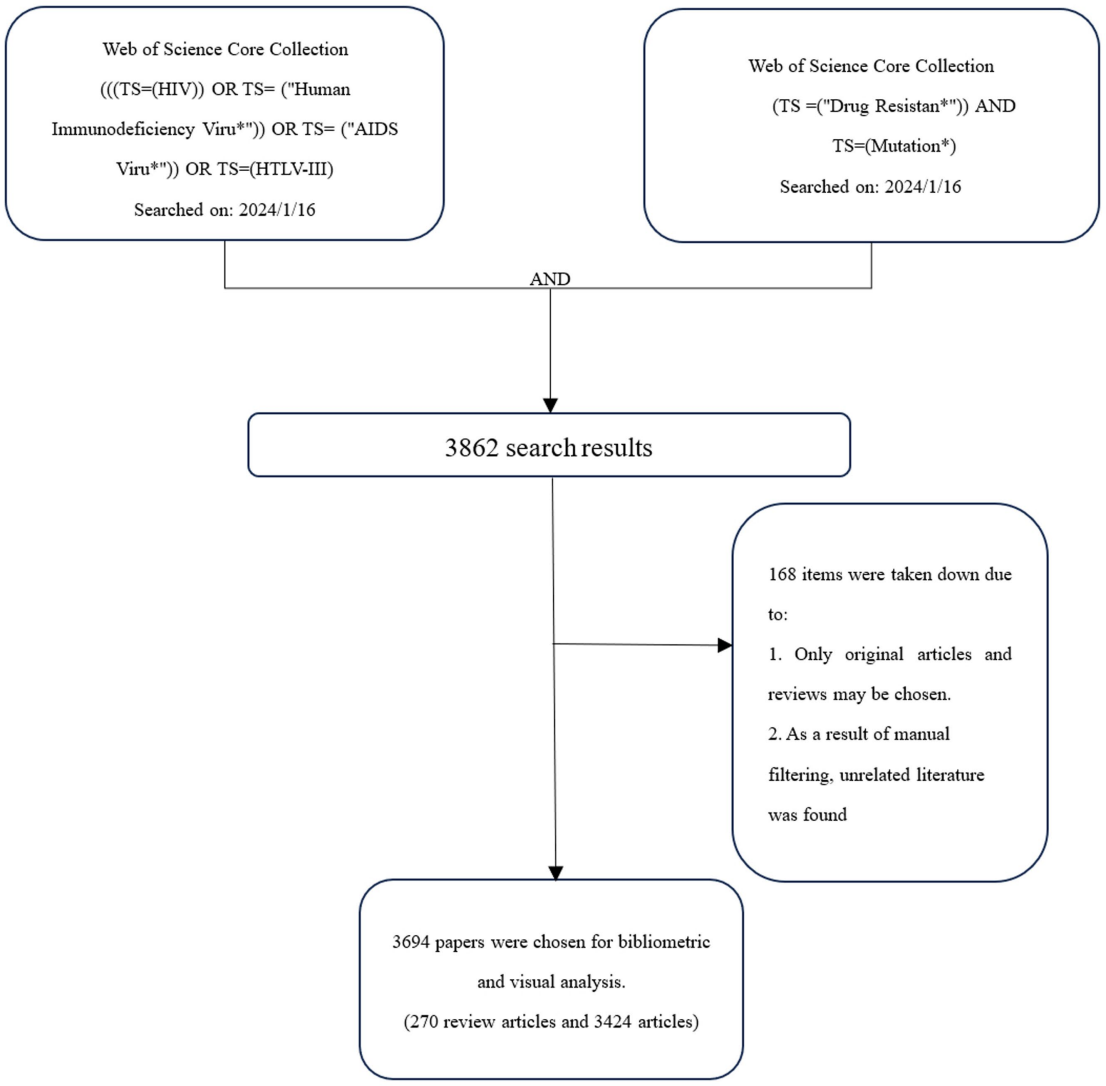
Retrieval process.

A diagram of the retrieval process is shown in Figure 1. According to the search rules of WOSCC “TS” represents the topic, in the search formula. The study period was January 1, 2011, through December 31, 2023. CiteSpace version 6.2.R7 was employed. Because the initial data obtained contained unrelated content, such as DRM data, all three researchers independently assessed the data from WOSCC and omitted unnecessary information and duplicate articles with CiteSpace’s “remove duplicates” feature and manual screening. The data were gathered on January 16, 2024, to offset the effects of the search library upgrade on this work.

### 2.2 Data export

The remaining 3,694 articles were then exported via WOS in the format of “full records and cited references” after removing any literature unrelated to the topic. Data from the first to the 500th place were imported into a file called “download_1-500.txt,” and data from the 501st to the 1000th place were imported into a folder named “download_501–1,000”; all data were exported in accordance with this pattern until the 3,694 articles were obtained.

### 2.3 Bibliometrics and visualization analysis

After eliminating duplicates and importing all WOSCC data into CiteSpace (version 6.2. R7), the data were converted to a format for visual analysis. CiteSpace utilizes nodes to indicate information such as country, institution, author, and keywords. In the graph for visual analysis from January 1, 2011, to December 31, 2023, “Years Per Slice” is set to 1 year. The frequency and year of occurrence are shown by the size and color of the nodes, respectively. The node radius increases with increasing total frequency of the node. The line between nodes represents the strength of the cooperation or occurrence between two nodes. Nodes with a red border are typically thought to have high centrality (centrality > = 0.1), making them hot research topics and key positions in the field (Fu et al., 2024).

## 3 Results

### 3.1 Analysis of the annual output of publications

**Figure 2:**
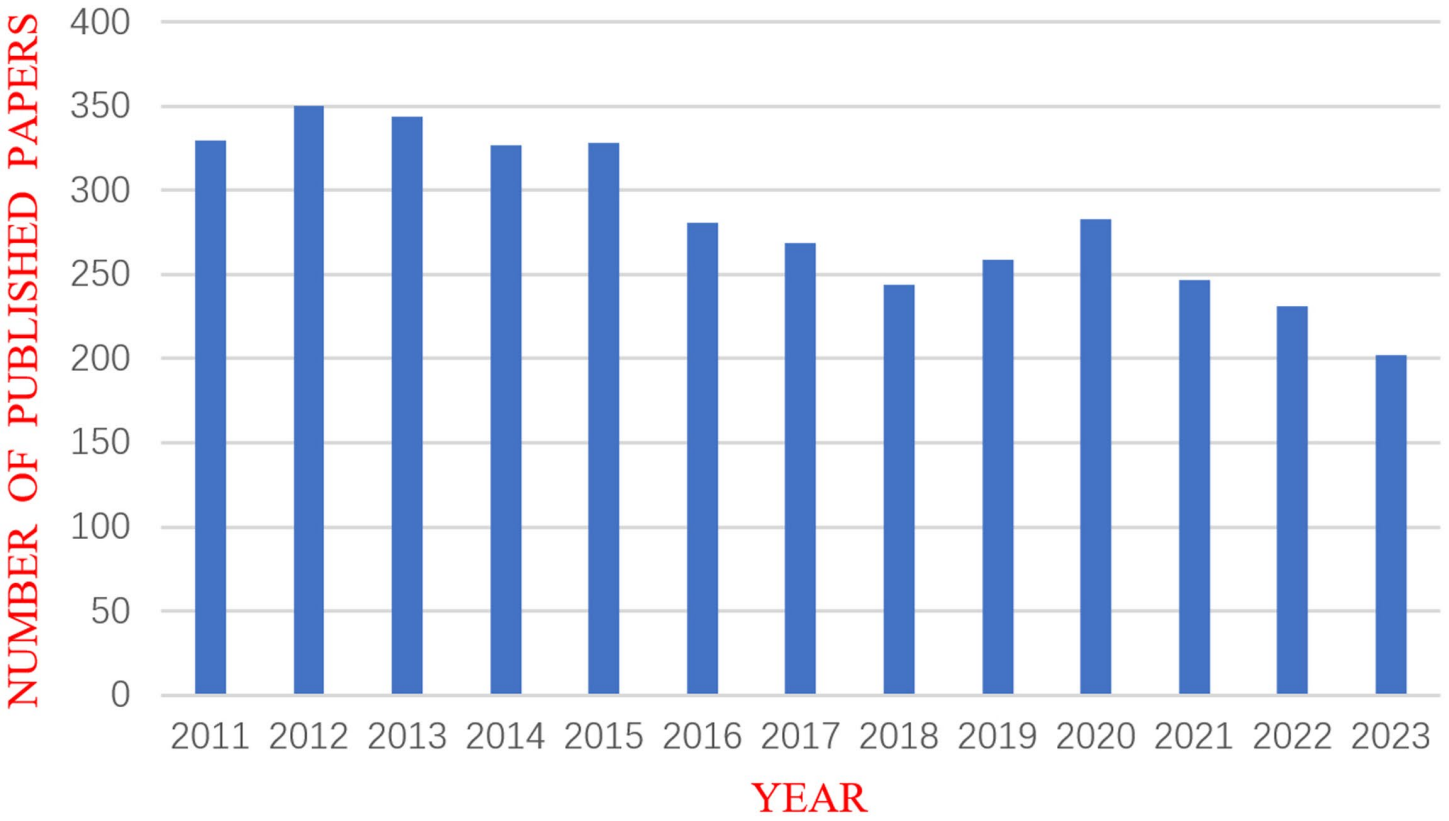
Annual chart of publications.

The quantity of academic papers published each year can provide insight into this field’s past and forecast its future growth and decline. After using CiteSpace to eliminate duplicates, the number of published years was used as the horizontal axis and the number of published articles as the vertical axis to create a chart for the annual outputs of publications, as shown in Figure 2. This figure demonstrates that the amount of DRM literature fluctuated over time. The year with the fewest articles published was 2023 (202, 5.5%), whereas the number of publications peaked in 2012 (350, 9.5%). The initial paper on DRMs in the database was published by Kazanjian et al. (1998). Extensive research has been conducted on early drug-resistant strains. However, new drug resistance mutations are continuously found, and these events occur irregularly, causing fluctuations in the quantity of studies.

### 3.2 Analysis of countries and institutions

**Figure 3:**
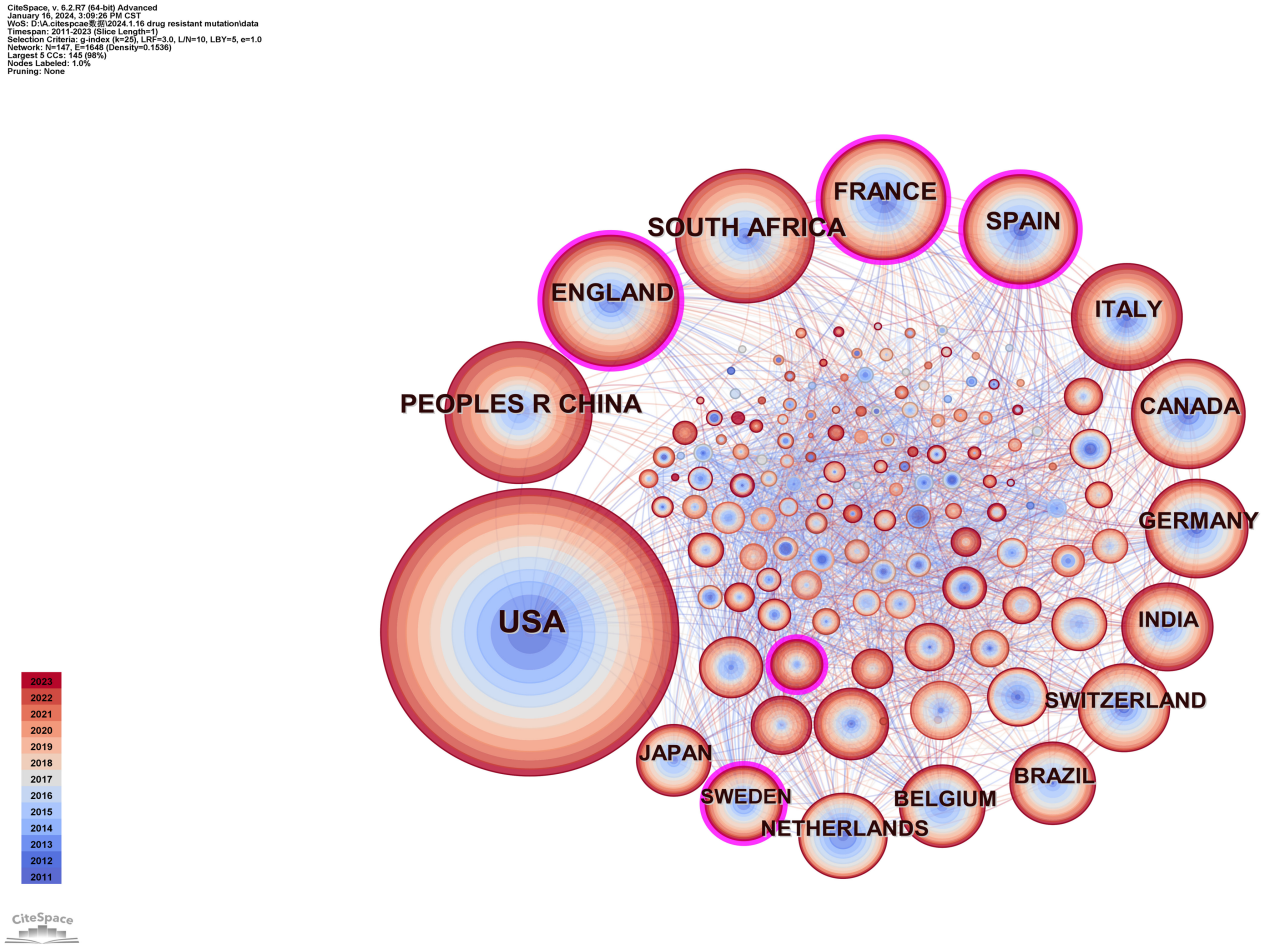
Map of countries.

**Figure 4:**
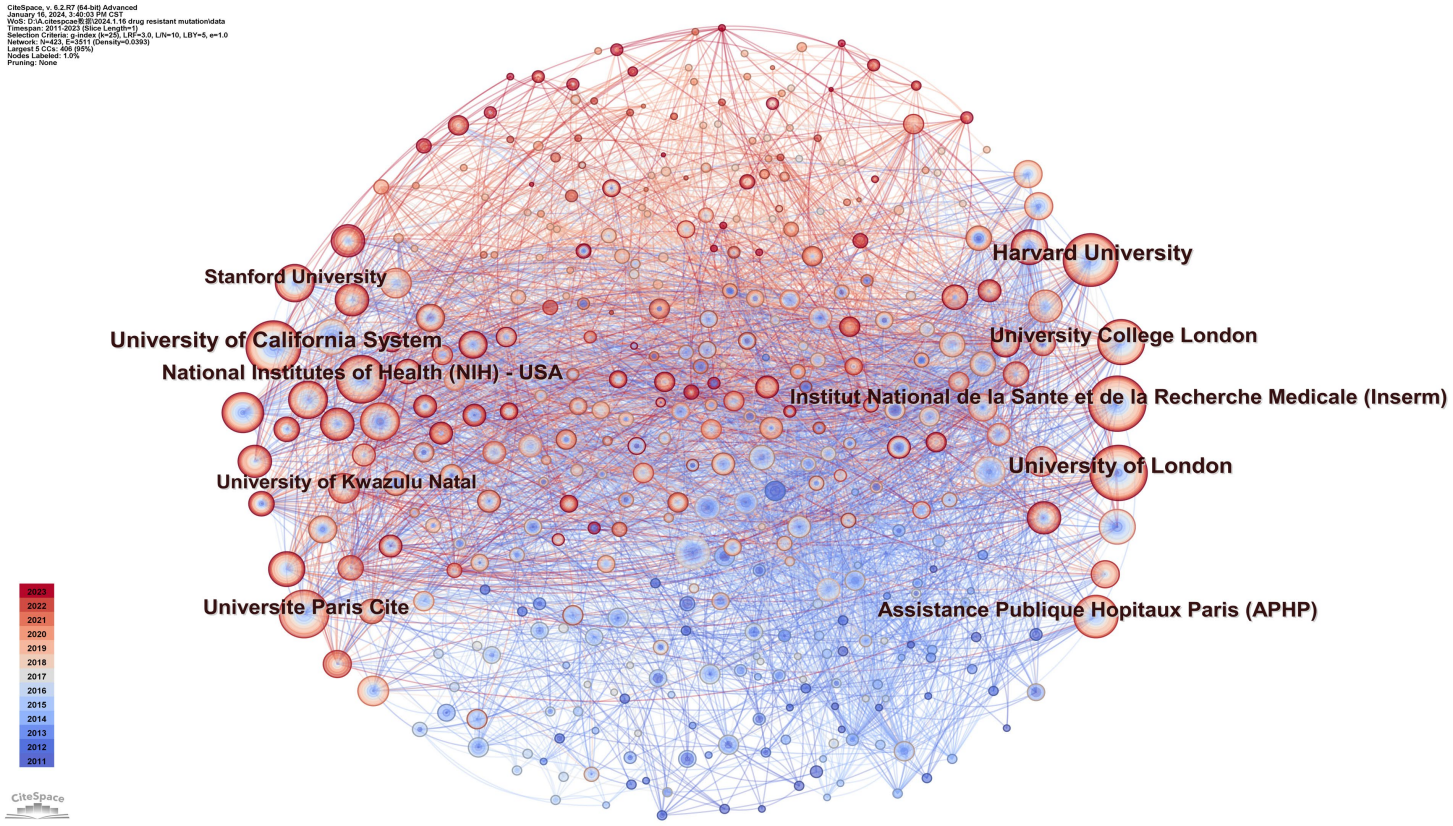
Map of institutions.

Using CiteSpace, we constructed a network diagram of countries and institutions to examine their level of cooperation. The top 10 countries and institutions are shown in Table 1. The top five nations were the USA (1,449, 39.2%), People’s Republic of China (411, 11.1%), South Africa (357, 9.7%), England (336, 9.1%), and France (298, 8.1%). The University of London (193, 4.9%), the University of California system (186, 5.0%), Harvard University (185, 5.0%), the Institut National de la Sante et de la Recherche Medicale (173, 4.7%), and Universite Paris Cite (162, 4.4%) were the top five institutions with the most publications. Figure 3 depicts a national cooperation diagram that includes 147 nodes and 1,648 connections, indicating close cooperation among countries, as represented by the United States. England, France, Spain, Sweden, and Honduras are shown as nodes with purple outlines, signifying their great centrality. With 423 nodes and 3,511 connections, the network map of institutional cooperation in Figure 4 illustrates tight collaboration between the institutions, as represented by the University of London.

**Table 1:** Top 10 countries and institutions with published articles.

Rank	Countries	Count	Institution	Count
1	USA	1,449	University of London	193
2	PR CHINA	411	University of California System	186
3	SOUTH AFRICA	357	Harvard University	185
4	ENGLAND	336	Institut National de la Sante et de la Recherche Medicale (Inserm)	173
5	FRANCE	298	Universite Paris Cite	162
6	SPAIN	250	National Institutes of Health (NIH) - USA	160
7	ITALY	240	Assistance Publique Hopitaux Paris (APHP)	146
8	CANADA	236	University College London	142
9	GERMANY	216	Stanford University	107
10	INDIA	172	University of Kwazulu Natal	105

### 3.3 Analysis of authors

**Figure 5:**
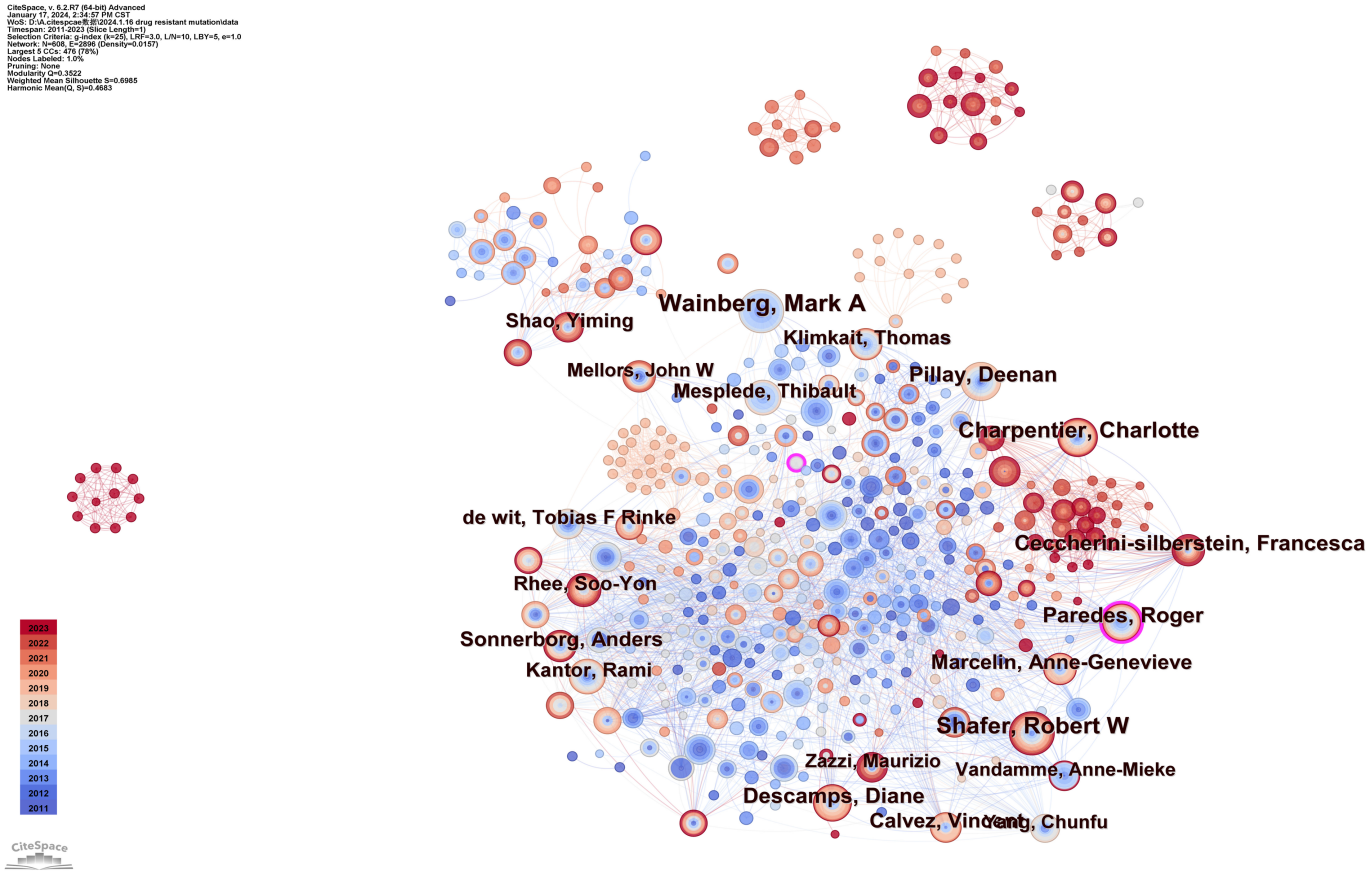
Map of coauthors.

To identify authors with high influence in DRM research, we created a coauthor network diagram with CiteSpace in which nodes represent authors and lines represent cowork relationships. The larger the radius is, the more articles the author posts. The bluer the color of the connection is, the earlier the first collaboration between authors is. As shown in Figure 5, there are 608 nodes and 2,896 connections in the map, with a stable cooperative relationship formed between researchers. The top 10 authors in the publications, as indicated in Table 2, produced 452 articles (12.2%). The most prolific author was Wainberg, Mark A., who contributed 66 articles (1.79%), followed by Shafer, Robert W. (58, 1.57%), and Charpentier, Charlotte (48, 1.30%). Paredes and Roger had the highest centrality (0.13) and strong cooperative relationships with other researchers. Nevertheless, the centrality of the nodes of these high-yield authors was low, Which indicated they and other researchers established more cooperation and communication to produce better research findings.

**Table 2:** Top 10 authors in publishing papers on drug resistance mutations.

Rank	Authors	Count	Centrality
1	Wainberg, Mark A	66	0.03
2	Shafer, Robert W	58	0.06
3	Charpentier, Charlotte	48	0.06
4	Pillay, Deenan	44	0.04
5	Paredes, Roger	43	0.13
6	Descamps, Diane	42	0.02
7	Ceccherini-Silberstein, Francesca	40	0.10
8	Marcelin, Anne-Genevieve	37	0.03
9	Sonnerborg, Anders	37	0.06
10	Calvez, Vincent	37	0.01

### 3.4 Analysis of reference data

**Figure 6:**
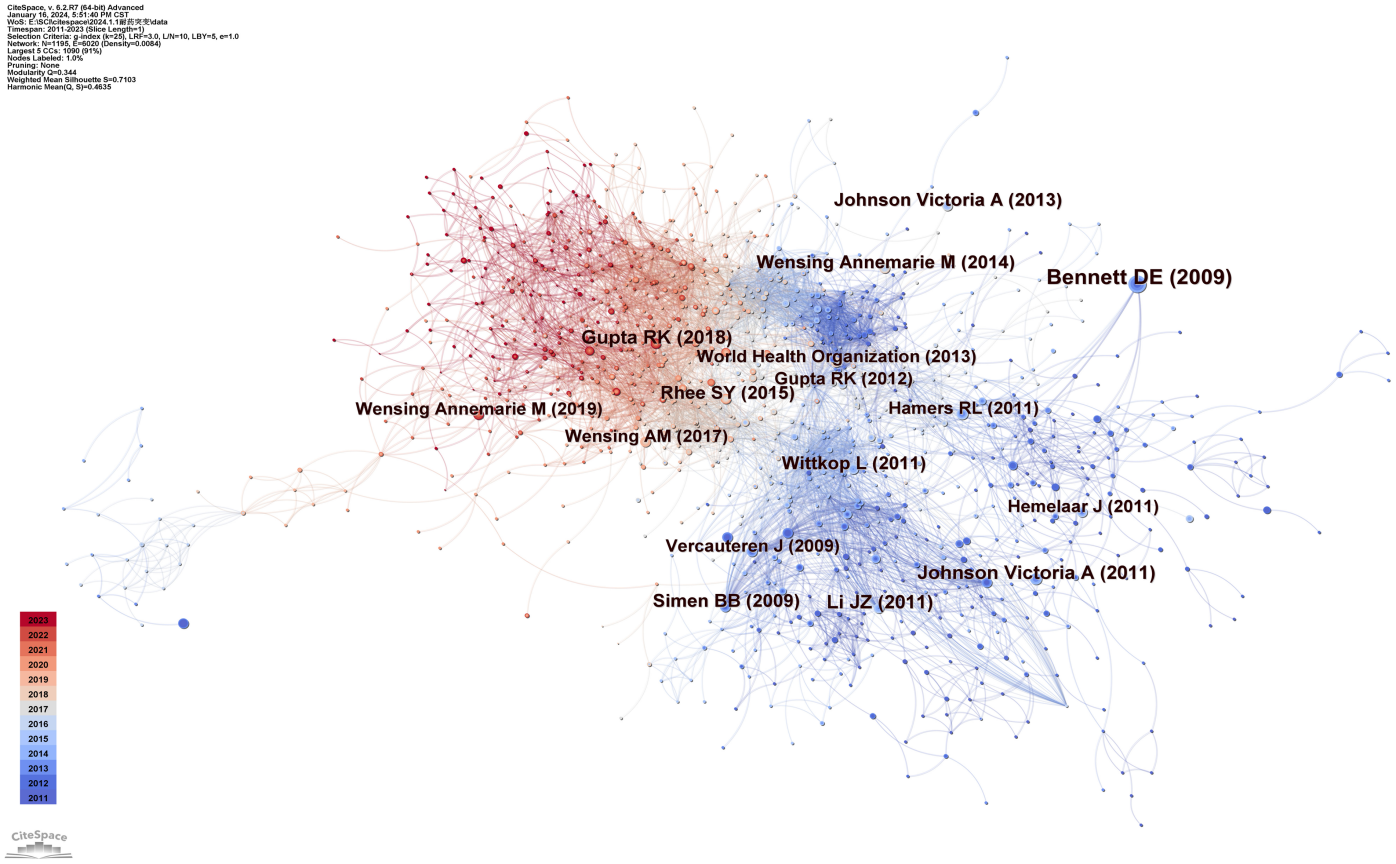
Map of cocited references.

There are 1,195 nodes and 6,020 lines in the drug resistance mutation map from cocited references in Figure 6, and Table 3 lists the top 10 references. When two pieces of literature are cited at the same time, this can be referred to as cocitation. The cocitation connection is considered the cornerstone for the foreseeable future. The most referenced study was published by Bennett et al. in 2007. This study updated the criteria for surveillance of drug resistance mutations (SDRMs), ranging from 80 mutations in RT and protease in 2007 to 93, which included NRTI resistance mutations, NNRTI resistance mutations, and PI resistance mutations (Bennett et al., 2009). This work contributed to the unified genetic definition of TDR, which aided in monitoring infections on regional, national, and local scales; supported treatment recommendations; and provided feedback on prevention projects. Gupta et al. conducted a meta-regression study with 358 datasets encompassing 56,044 people in 63 countries regarding pretreatment drug resistance (Gupta et al., 2018). It is an assessment of drug resistance in low- or middle-income nations in which ART is initiated or restarted in HIV-1 patients as first-line therapy. This study demonstrated how rapidly pretreatment drug resistance is spreading in these countries, particularly in sub-Saharan Africa. The incidence of NNRTI pretreatment resistance is almost at the WHO threshold. These findings suggest that long-term adoption of national HIV medication resistance tracking is necessary and that first-line ART strategies require revision. Johnson et al.'s, 2011 update on HIV-1 resistance mutations is the third most cited piece of literature (Johnson et al., 2011). This paper adds to the literature regarding HIV-1 resistance for future studies.

**Table 3:** Top 10 cited references of drug resistance mutations.

Rank	Cite Reference	Count	Centrality
1	Bennett DE, 2009, PLOS ONE, V4, P0, DOI 10.1371/journal.pone.0004724	220	0.05
2	Gupta RK, 2018, LANCET INFECT DIS, V18, P346, DOI 10.1016/S1473-3099(17)30702-8	123	0.17
3	Johnson Victoria A, 2011, TOP ANTIVIR MED, V19, P156	115	0.04
4	Li JZ, 2011, JAMA-J AM MED ASSOC, V305, P1327, DOI 10.1001/jama.2011.375	111	0.14
5	Wittkop L, 2011, LANCET INFECT DIS, V11, P363, DOI 10.1016/S1473-3099(11)70032-9	107	0.10
6	Rhee SY, 2015, PLOS MED, V12, P0, DOI 10.1371/journal.pmed.1001810	100	0.30
7	Wensing Annemarie M, 2014, TOP ANTIVIR MED, V22, P642	97	0.00
8	Simen BB, 2009, J INFECT DIS, V199, P693, DOI 10.1086/596736	95	0.03
9	Johnson Victoria A, 2013, TOP ANTIVIR MED, V21, P6	88	0.01
10	Vercauteren J, 2009, J INFECT DIS, V200, P1503, DOI 10.1086/644505	88	0.03

### 3.5 Analysis of journals: overlay map

**Figure 7:**
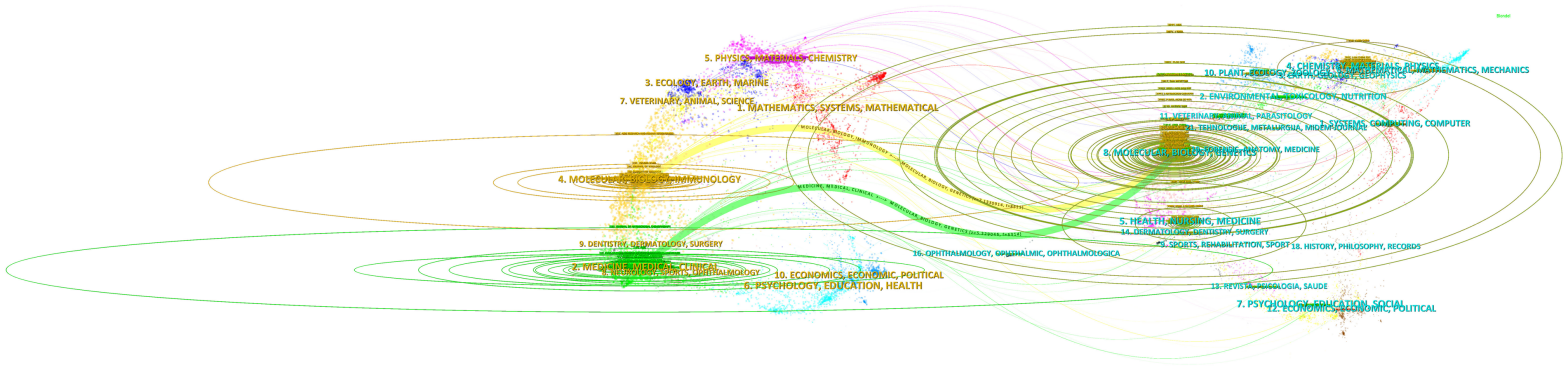
Overlay map of journals.

Figure 7 was created using CiteSpace’s “overlay maps” tool. The citing journals on the left and the cited journals on the right illustrate the links between them. The curve shows the citation relationship. The elliptic curve reflects the proportion between authors and publications. The more writers there are, the longer the ellipse’s horizontal axis is; as the vertical axis of the ellipse lengthens, more papers are published in the journal. Medical and clinical systems, molecular biology, immunology, psychology, education, health, neurology, sports, ophthalmology, dentistry, dermatology, surgery, and economics were the key research sources for this subject. Publications related to environmental, toxicology, nutrition, systems, computing, health, nursing, medicine, psychology, education, social, ophthalmology, and ophthalmic topics were widely cited. This suggests that the problem of medication resistance mutations is tied to psychology, education, economics, immunology, and clinical medicine.

### 3.6 Analysis of keywords

#### 3.6.1 Analysis of keyword co-occurrence

**Figure 8:**
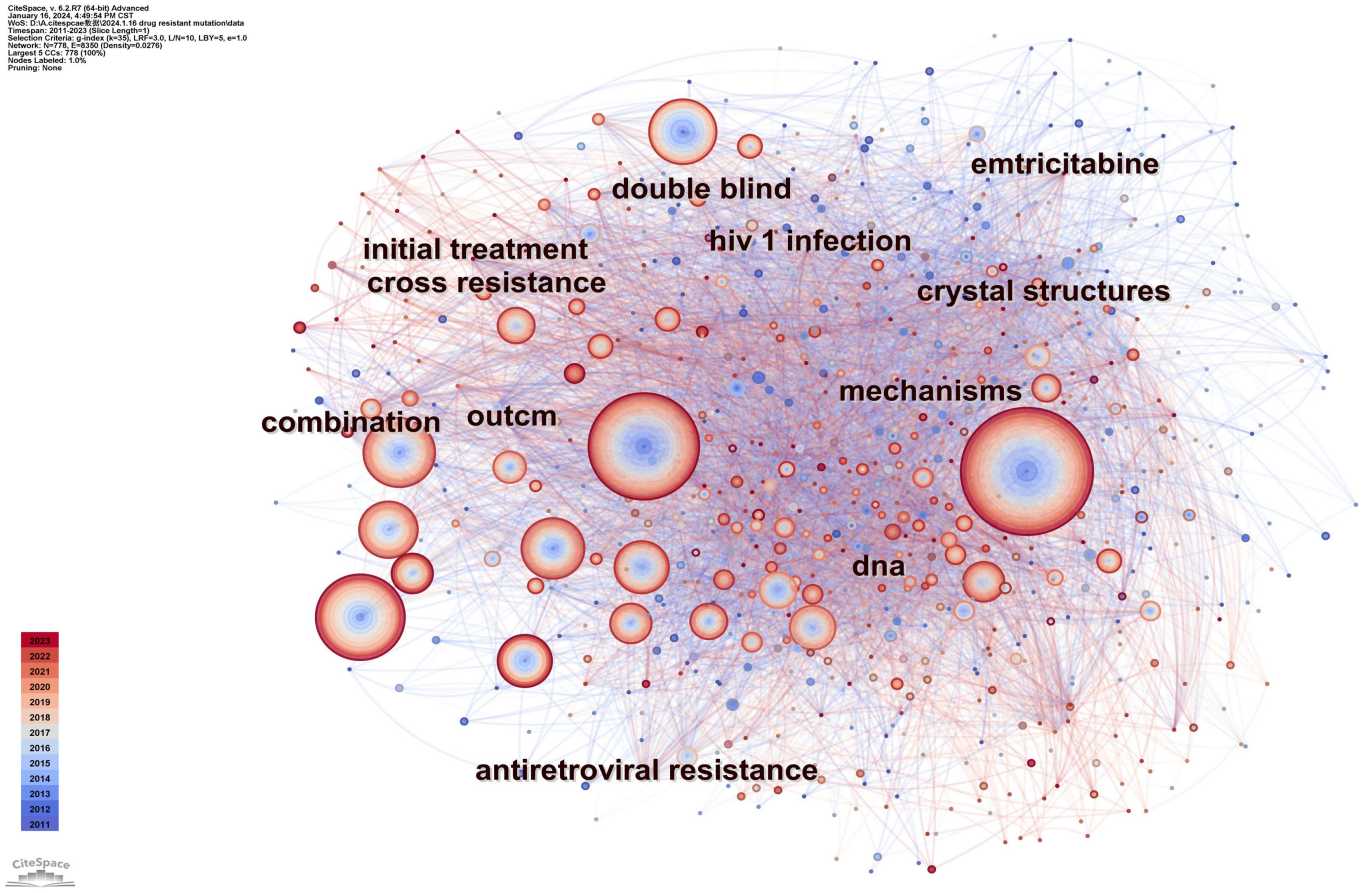
Map of keyword co-occurrence.

Keyword analysis emphasizes a field’s research output and depicts its hot topics and frontiers at a given time. Table 4 displays the top ten terms with the most occurrences. The most prevalent occurrence was drug resistance (frequency: 1383), followed by mutations, antiretroviral therapy, reverse transcriptase, and HIV-1. Figure 8 displays a CiteSpace keyword co-occurrence map with the g-index set to 35. There are 8,350 lines and 778 nodes in this map. The older the relationship between two nodes is, the bluer the line is.

**Table 4:** Top 10 reference keywords related to drug resistance mutations.

Keywords	Count	Centrality
Drug resistance	1,383	0.00
Mutations	1,017	0.01
Antiretroviral therapy	707	0.01
Reverse transcriptase	553	0.01
Immunodeficiency virus type 1	500	0.01
Prevalence	447	0.01
Infection	425	0.01
Therapy	383	0.02
Human immunodeficiency virus	348	0.01
Transmission	301	0.01

#### 3.6.2 Analysis of keyword clusters

**Figure 9:**
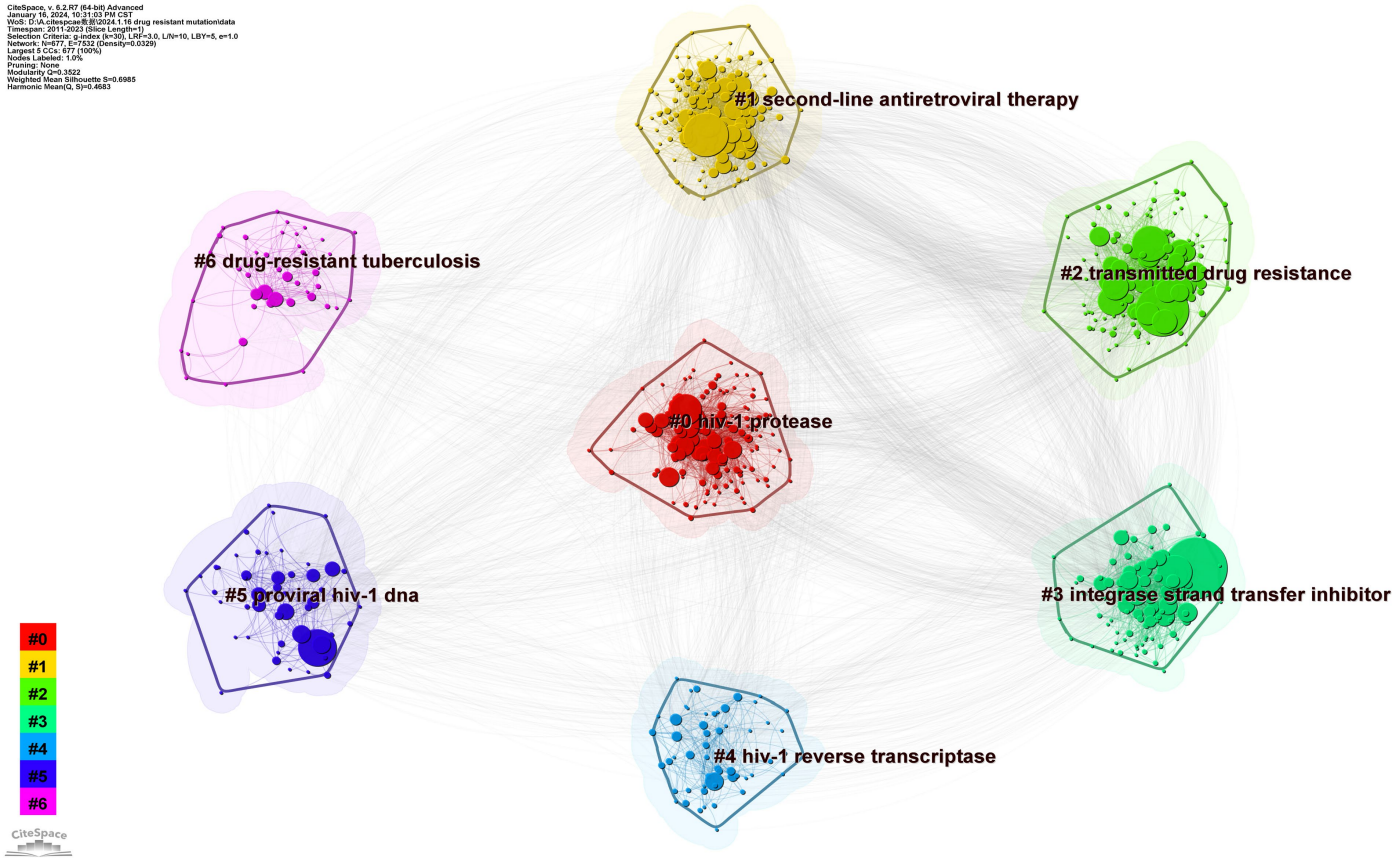
Map of keyword clusters.

We set the factor k to 30 by conducting a cluster analysis based on keyword co-occurrence. The log-likelihood radio in CiteSpace typically produces the best cluster results. The seven clusters of HIV-1 protease, second-line antiretroviral therapy, transmitted drug resistance, integrase strand-transfer inhibitor, proviral HIV-1 DNA, drug-resistant tuberculosis, and HIV-1 reverse transcriptase are depicted in Figure 9. The clustering result is considered reasonable if the average contour value S is larger than 0.5 and effective if it is greater than 0.7. The clustering module value Q is crucial for evaluating rationality indicators. When Q is greater than 0.3, the cluster community structure is significant (Chen, 2006). Our clusters are effective, according to the results of Q (Q = 0.3522) and S (S = 0.6985) of keyword clusters mapped by topic with drug-resistant mutations. By summarizing the characteristics of these clusters, they can be divided into four topics: type of inhibitors, challenges in preventing and spreading, modifications to therapeutic approaches, and information about coinfection.

##### 3.6.2.1 Types of inhibitors

An academic circle’s focus has resulted in the appearance of three connected clusters on our map, which are HIV-1 protease (#0), integrase strand-transfer inhibitor (#3), and HIV-1 reverse transcriptase (#4), representing the four types of inhibitors in PIs, INSTIs, NRTIs, and NNRTIs, respectively.

HIV protease (PR) is the only protease of the virus that performs proteolysis of gag and gag-pol into all structural proteins (Ghosh et al., 2016). Therefore, numerous drugs, called PIs, have been developed. Most PIs that mimic the natural substrate of PR initially inhibit PR binding sites through competitive means (Wensing et al., 2010). Two generations of PIs, namely, indinavir, nelfinavir, lopinavir, and tipranavir, have been developed since saquinavir was first identified in 1995 (Ghosh et al., 2016). However, since PIs were first used in clinical settings, there is an opportunity for severe resistance mutations to be produced with this medication. According to a recent publication on PI mutations, the most prevalent mutation types are V82ACS, M46IL, 154MTV, and L76V. After receiving PI treatment for an extended period, one-third of patients develop significant resistance to the drug (Chimukangara et al., 2022). Consequently, although PIs are considered to have a high genetic barrier, more studies and advancements are needed for patient drug resistance testing.

By chelating divalent metal cations at the catalytic site of the enzyme, INSTIs block HIV-1 integrase, preferentially binding to the the active site of integrase with its cofactor over free integrase (Craigie, 2001; Zhao et al., 2022). The emergence of elvitegravir, bictegravir, dolutegravir, and raltegravir signifies that more substantial genetic barriers in INSTIs have been discovered (Max, 2019; Zhao et al., 2022). Clinical treatment is less potent when multiple mutations coexist, and the susceptibility to all INSTIs is reduced by more than 300 times (Cheung et al., 2022). Research has also revealed the side effects of INSTIs. Dolutegravir (DTG), for instance, has been linked to weight gain, neural tube abnormalities, and neuropsychiatric disorders (Scévola et al., 2020). Patients receiving elvitegravir and cobicstat may experience fatigue, malaise, and gastrointestinal symptoms (Lepik et al., 2018). As a result, we must consider how patients with infection and numerous mutations are treated and how these people are impacted by the side effects of these drugs, especially DTG.

NNRTIs and NRTIs inhibit viral replication by targeting the HIV reverse transcriptase process (Yoshida et al., 2021). Although NNRTIs are thought to be safe and well-tolerated medicines, increasing evidence suggests that they may be linked to gastrointestinal toxicity, skin responses, neuropsychiatric symptoms, hepatotoxicity, and metabolic abnormalities (Blas-Garcia et al., 2011; Reust, 2011). For NRTIs, some RT mutations, such as M184V, L74V, Q151M, and K65R, can lead to binding evasion while retaining the ability to recognize natural substrates; other mutations, such as M41L, D67N, K70R, cause enhanced phosphorolytic removal at the 3′-terminus of the primer after viral DNA binding by NRTIs, with others resulting in removal to overcome chain termination (Marcelin, 2006). Numerous novel chemicals and drug candidates have been created recently to address the problems of drug resistance and adverse reactions. As potent inhibitors of the novel HIV-1 NNRTI, diarylpyrimidines have been extensively synthesized in a variety of configurations (Huang et al., 2019; Liu et al., 2019). More investigations are required to develop more modern medications based on this kind of medication.

##### 3.6.2.2 Challenges of prevention and spread

Pertaining to clusters 2 and 5, the presence of proviral DNA and spread of medication resistance may pose challenges to HIV control and treatment.

Proviral DNA, which is surrounded by two lengthy terminal repeats, is generated by copying of genomic RNA by reverse transcriptase (Mougel et al., 2009). ART cannot eradicate cells in which HIV-1 previral DNA is incorporated, which can act as a cell reservoir to store this DNA. As a result, these cell reservoirs may reseed the virus for reproduction when ART is discontinued (Linden and Jones, 2022). Since memory T cells have a lengthy half-life, the proviral DNA of the virus that has been integrated into CD4 T cells may retain drug resistance-related mutations. This attribute renders it an ideal target for the precursor DNA reservoir (Turriziani et al., 2010; Geretti et al., 2022). Therefore, one of the significant obstacles to curing HIV is the presence of drug-resistance wild-type previral DNA in memory T cells.

Drug resistance can be precisely detected, and its extent of distribution can be determined by deep sequencing. Transmission of drug resistance occurs when an HIV-1 strain acquires one or more mutations linked to treatment resistance. Recent reports on global surveillance of HIV-1-transmitted drug resistance have shown that NNRTI and NRTI resistance is increasing in sub-Saharan Africa, NNRTI resistance is increasing in Latin America and the Caribbean, and NNRTI and PI resistance is generally increasing in North America (Rhee et al., 2020). This result demonstrates the importance of publishing study findings and monitoring global TDR. Ragonnet-Cronin and colleagues proved that clinical baseline diagnostic serum genotyping can be used for HIV TDR surveillance of treatment-naïve individuals (Ragonnet-Cronin et al., 2013) This achievement supports ongoing global TDR monitoring. Nichols et al. discovered a negative correlation between viral load and CD4+ T-cell count decline using ultradeep sequencing to evaluate the presence of minority patient TDR. This means that negative selection will affect a small percentage of the viral population’s transmission resistance (Nichols et al., 2014). This phenomenon is intriguing and merits further attention. The strains found in these samples may help in determining how to choose a drug for treatment-naïve individuals. Given that TDR may have an impact on antiretroviral therapy (Aghokeng et al., 2011), it is essential to monitor DRM status by deep sequencing while providing feedback to alter the first-line treatment plan and reduce HIV selection and transmission. Notably, the two studies mentioned above were carried out in settings with limited resources. As a result, more relevant studies in affluent areas must be conducted to obtain more persuasive results.

##### 3.6.2.3 Modifications to therapeutic approaches

This section is mainly related to cluster 1. In cases in which first-line treatment fails virologically, second-line therapy—typically consisting of a PI combined with two NRTIs—is implemented (Hosseinipour et al., 2013). According to a related investigation, low adherence leads to severe treatment failure in locations with few resources (Ajose et al., 2012). Indeed, low adherence may be one factor contributing to second-line therapeutic failure, according to research performed by Mantshonyane (Mantshonyane et al., 2021). The index of first-line therapy adherence serves as a predictor of second-line therapy adherence (Ramadhani et al., 2014). Furthermore, it is thought that a patient’s weight may be associated with virologic failure (Salvi et al., 2022). If second-line therapy fails, third-line therapy needs to be attempted. One such medication combination that has been proven to be well tolerated is raltegravir, darunavir, and etravirine (Avihingsanon et al., 2022). Additionally, a better second-line alternative is a wise decision for clinical trials (Keene et al., 2021). Thus, prior to therapy, it is necessary to improve patient compliance. Further research is needed to determine the association between weight and virological failure. Future studies on better medicine combinations also need to be carried out.

##### 3.6.2.4 Resistance to HIV coinfected viruses

This section is associated with cluster 6. This cluster’s form may be caused by the coinfection rate and severe mortality rate. When HIV and tuberculosis (TB) co-occur, the situation becomes more complex. Standard first-line treatment is virtually unavoidable for multidrug-resistant TB, which is resistant to isoniazid and rifampicin (Seung et al., 2015; Singh et al., 2020). Coinfection increases the rate at which TB progresses and increases the spread of drug-resistant TB (Khan et al., 2019). According to research by Anderson et al. on the mortality of drug-resistant TB patients, individuals with coinfections had an average 1.5-year shorter survival period than those with single infection (Anderson et al., 2022). The following coinfective variables are associated with mortality: BMI, mid-upper arm circumference, use of bedaquiline therapy, use of ART, and detectability of viral load (Bayowa et al., 2023). Moreover, coinfections increase the frequency of side effects such as depression, nephrotoxicity, and hearing loss (Lazarus et al., 2021). For patients who are coinfected, medical personnel should focus on these indices. Notably, the metabolic effects of many medications cannot be disregarded because HIV patients frequently receive multiple pharmacological treatments. Isoniazid combined with NNRTIs and PIs, for example, may increase associated toxicities (Mukonzo et al., 2019). Thus, additional research on the interactions between medications used to treat HIV and TB is needed. Furthermore, healthcare providers should avoid combining medications in a way that compromises drug metabolism.

##### 3.6.2.5 Analysis of variation in clusters

**Figure 10:**
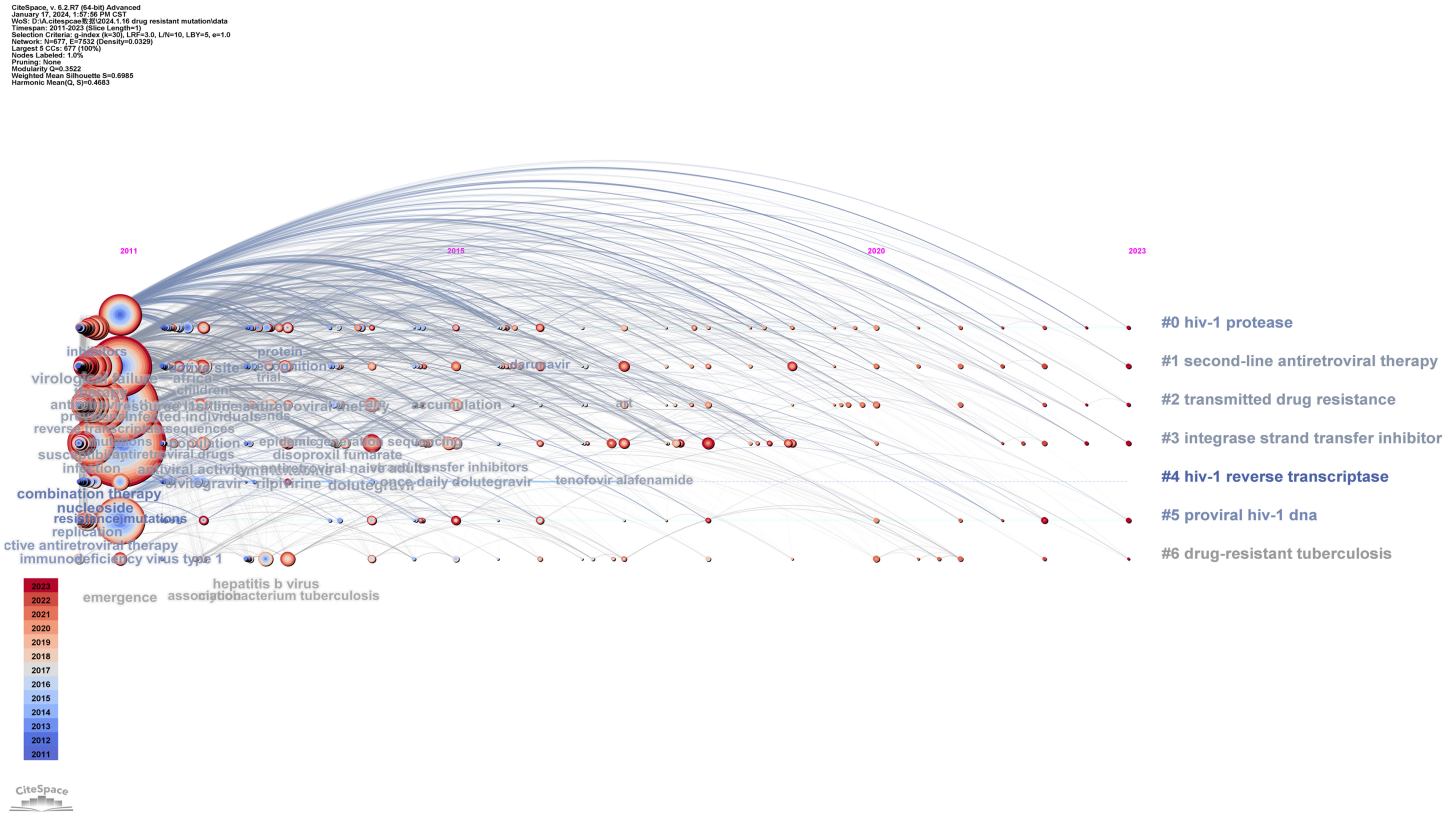
Timeline view of the keyword co-occurrence map.

**Figure 11:**
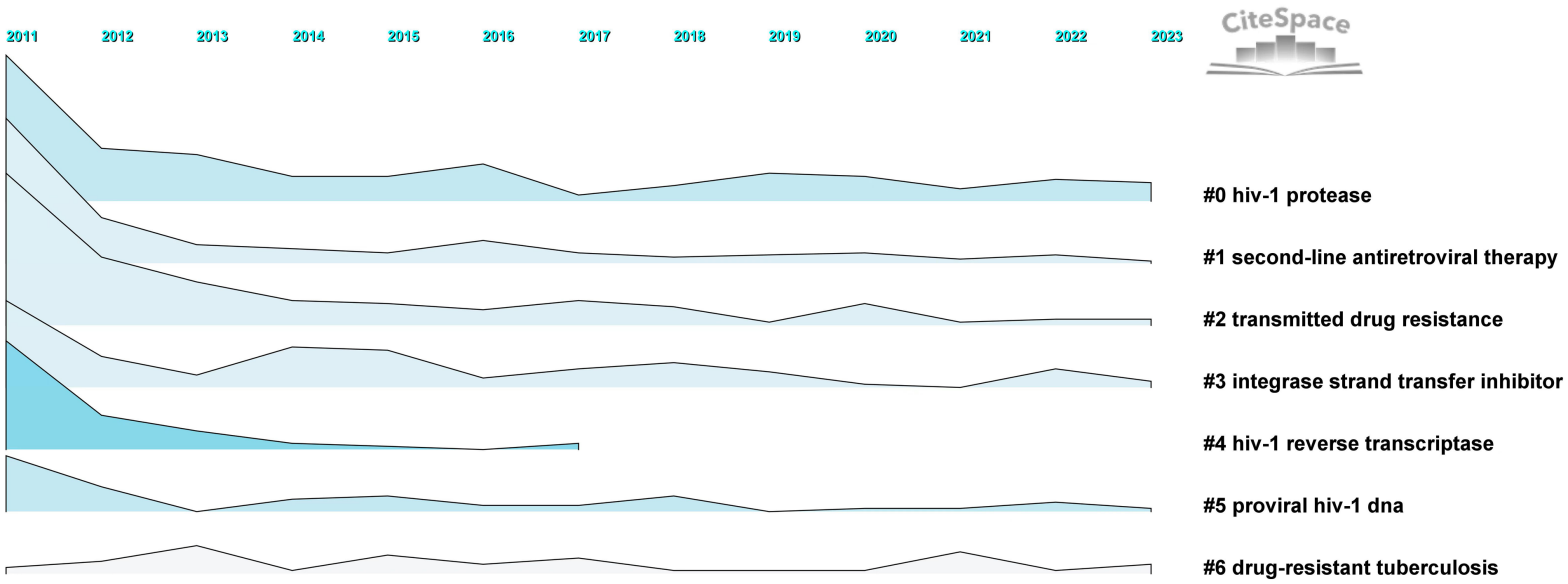
Landscape of clusters.

The results of timeline and landscape view analyses are shown in Figures 10, 11, respectively, revealing how hot research topics change over time and aiding in comprehending the evolution of the field. The largest cluster in our study, cluster 0, indicates that protease, or PI, is consistently the main focus of academic circles. Investigation of medications and their underlying mechanisms remains a primary focus, and steady advancements have been made. Cluster 2 had a low update status for the past 3 years, which might mean that the spread of medicine resistance has been limited. Cluster 3 displays a rise in keywords between 2021 and 2022, suggesting recent advancements in the subject. Little change in the coinfection resistance of pulmonary TB has been observed, revealing that further research in this field is necessary. Since there have been no recent new keywords for cluster 4, further innovation in this area is needed. The number of keywords in other clusters has increased recently, implying that these disciplines have been steadily developing. Furthermore, controlling HIV infection remains a challenging task despite technological advancements. In addition, INSTI drugs have been extensively used in clinical treatment to reduce HIV replication, resulting in countless clinical reports that have produced a wealth of novel findings, including mutations, side effects, and development of related, more potent new drugs.

#### 3.6.3 Keywords with citation bursts

**Figure 12:**
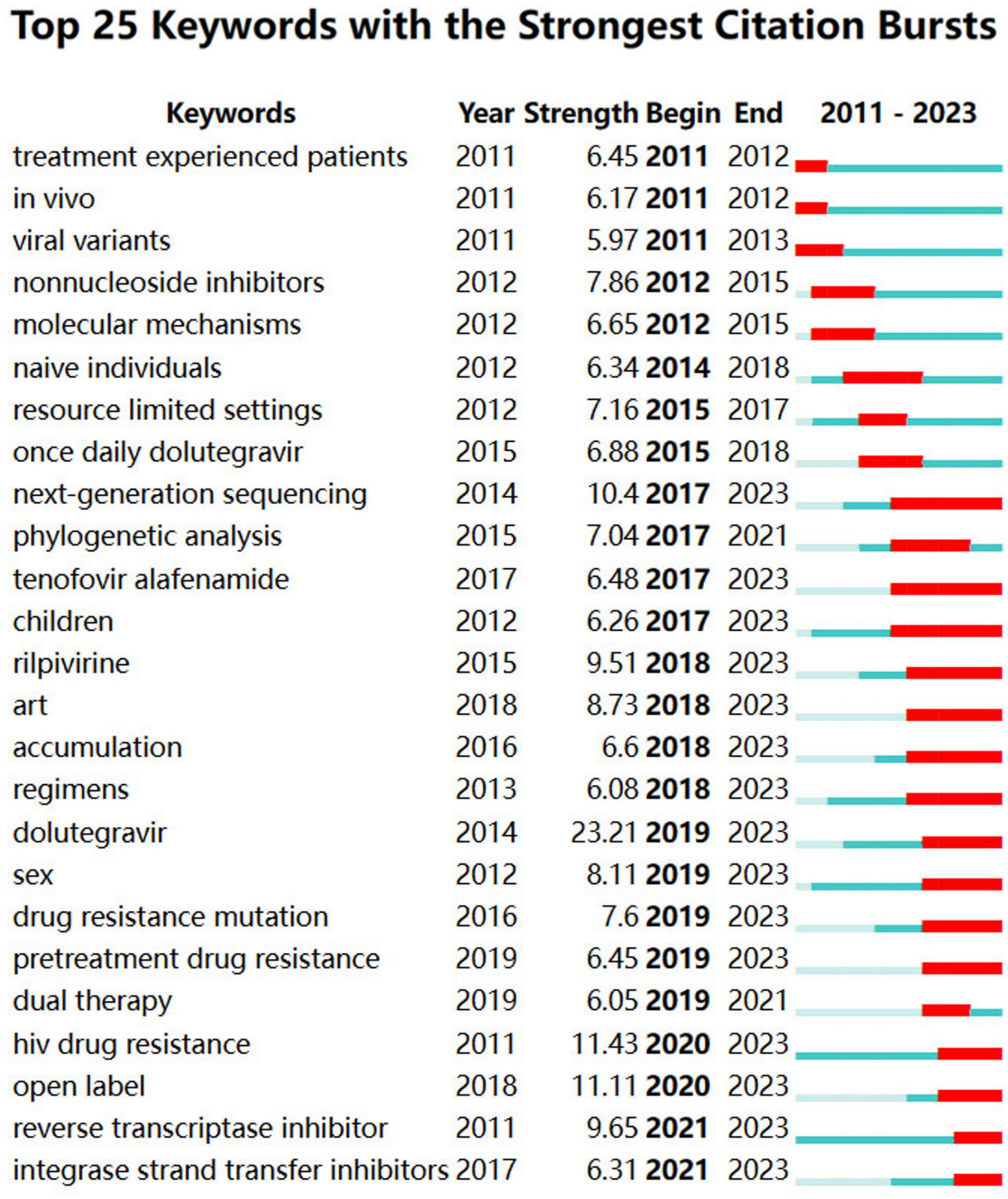
Top 25 keywords with the strongest citation bursts.

Citations with keywords surged in several time intervals, signifying a strong focus in the academic cycle. Hence, concentrating on keyword bursts is conducive to straightforwardly grasping research frontiers and trends. The top 25 related DRM terms, as determined by burst detection methods with the highest citation bursts, are shown in Figure 12. The blue line denotes the time interval, whereas the red line denotes an interval period with a keyword burst. The word “dolutegravir” is the most vital intensity term in this research, as shown in Figure 12. It has been growing rapidly since 2019 and continues to grow, suggesting that DTG is an effective substitute for first-line resistance inhibitors. In recent years, there has been a significant level of burst intensity surrounding the terms “next-generation sequencing,” “tenofovir alafenamide,” “children,” “regimens,” “accumulation,” “dolutegravir,” “rilpivirine (RPV),” “pretreatment drug resistance,” and “open label,” which are the frontiers in the DRMs area. They can be separated into four categories: crowd, research, drug, and hindrance. The following sections describe their traits in detail. We have already discussed the content of “reverse transcriptase inhibitors,” “integrase strand transfer inhibitors,” and modifications to clinical therapy regimens. In addition, “ART” is usually considered the abbreviation for antiretroviral therapy, and “HIV drug resistance” and “drug resistance mutation” are comprehensive concepts that are not covered in the analysis that follows.

##### 3.6.3.1 Frontier information of drugs

The burst results reveal that tenofovir alafenamide (TAF), DTG, and RPV are the three most potent medications. TAF functions by obstructing the catalytic site of HIV reverse transcriptase (Li et al., 2022), which has been the initial therapeutic option for HIV-1 (Ueaphongsukkit et al., 2021). Although TAF, the prodrug of tenofovir, is thought to have superior effects on bone mineral density and kidney function, subsequent studies have revealed that it can still exert some nephrotoxicity (Gupta et al., 2019; Ueaphongsukkit et al., 2021). Nevertheless, TAF-containing regimens are thought to provide safe and effective viral suppression for children and teenagers. This is a helpful reminder since there are few available drugs (Agwu et al., 2014; O'Rourke et al., 2023). As a tricyclic carbamoyl pyridone analog, DTG is absorbed and spread through cerebrospinal fluid and impairs the HIV integrase-DNA complex through chemical synthesis (Kandel and Walmsley, 2015). Antiretroviral therapy with two-drug regimens is practical and appealing owing to the decreased medium- and long-term toxic effects, its intricacy, and the associated expenses (Antinori et al., 2019). R263K, G118R, N155H, and Q148H/R/K are mutations associated with DTG, particularly G118R and R263K (Tao et al., 2023). Significant weight gain and neuropsychiatric symptoms are two of its related side effects (Kolakowska et al., 2019; Eckard and McComsey, 2020). RPV is the e-isomer of dapivirine and is found in the diarylpyrimidine dapivirine (Li et al., 2022). Due to its safety, RPV has been demonstrated to be the preferred choice for treatment-naïve patients, and the mutations that cause the RPV to lose effectiveness are K103N and M184I/V (Putcharoen et al., 2013). Nonetheless, derivatives developed based on RPV are an excellent choice for combating these mutations (Smith et al., 2016). Moreover, studies have shown that the therapeutic impact of rilpivirine with DTG and lamivudine with DTG is comparable to that of conventional three-drug regimens (Fida et al., 2020). Therefore, drug combination techniques relying on TAF should be widely used in clinical practice, and more studies on the adverse effects of TAF are necessary. There is potential for creating novel medications including RPV.

##### 3.6.3.2 Frontier population information

Because our map contains the term “children” as a burst, the drug resistance of these young people presents a novel potential situation requiring further exploration. NNRTI-based regimens were the initial choice for this group (Agwu et al., 2014). The number of adolescent HIV patients will increase as a result of the growing number of pediatric patients. There is less drug pressure due to restricted drug selection, which lowers the rate of mutation in this population (Agwu et al., 2014). Nevertheless, according to research from China, significant mutations affect approximately 73.1% of patients. NRTI, NNRTI, and dual-class resistance are responsible for 48.4, 63.4, and 38.7% of cases, respectively (Yan et al., 2022). This outcome demonstrates that because children have been treated for an extended period, the rate of mutations in this group has increased recently. A recent report on use of RPV for treatment of HIV-positive adolescents revealed that while the effectiveness of RPV decreased with prolonged use, its safety remained stable (Lombaard et al., 2022). In addition, TAF-containing regimens have been demonstrated to be a not-to-be-ignored strategy for children (O'Rourke et al., 2023). When second-line ART is begun in children, their CD4+ T lymphocyte count increases, and their mortality and loss to follow-up decrease (Collaborative Initiative for Paediatric HIV Education and Research (CIPHER) Global Cohort Collaboration, 2020). Consequently, treating drug resistance in children is positively impacted by prompt medication modifications. More research should be conducted on regimens to prevent children from becoming drug resistant.

##### 3.6.3.3 Frontier research information

The appearance of “open-label” and NGS indicates the research frontiers of DRMs. With its high-throughput, fast sequencing, next-generation sequencing has revolutionized genomic research (Levy and Boone, 2019). In contrast to classical Sanger sequencing, which detects substitutions, small insertions, and deletions, NGS does not require extra assays to collect mutation data; instead, mutation data are obtained through NGS. In addition, increasing the sequencing depth can increase the sensitivity of NGS (Behjati and Tarpey, 2013). With its high throughput for HIV-1, NGS can sequence full-length genomes while identifying HIV-1 genotypes with low levels of medication resistance (Metzner, 2022). In a high-throughput manner, NGS can detect 1% or fewer minor mutations, making it the first choice for HIV superinfection (Redd et al., 2013). Nonetheless, several of the steps of the NGS workflow, such as the sample type, PCR amplification, and initial material, are susceptible to bias. Moreover, there are difficulties with clinical care, especially in settings with few resources. It is challenging to popularize NGSs in these places because of infrastructure and equipment needs, cost, logistics, and supply chains (Ávila-Ríos et al., 2020). Furthermore, similar to previous sequencing platforms, the high throughput of NGS relies on both the amount and quality of the initial template input, meaning that NGS alone cannot identify low copy numbers in genomes (Munyuza et al., 2022). Therefore, further NGS optimization studies must be performed, as should maintaining the NGS supply chain and logistics in low-income areas and improving NGS usage training for medical personnel working in these locations.

After individuals complete the double-blind phase of the trial for a novel medication, they are offered the opportunity to participate in the open-label extension phase to obtain data on the drug’s long-term safety and tolerability (Taylor and Wainwright, 2005). Open-label studies differ from double-blind studies in that both the participants and investigators are aware of the true administration. For 96 weeks, an open-label, randomized, noninferiority trial examined the efficacy of therapeutic therapy or pharmacological combination virology in children and adolescents. Comparing the DTG group to the standard-care group, it was demonstrated that the former had reduced rates of death and virological failure, optimized CD4 count results, no drug-resistant mutations with first-line therapy, and fewer mutations with second-line therapy (Turkova et al., 2021). This finding indicates that DTG outperformed standard care in terms of inhibitory efficacy for younger patients. Open-label studies, as opposed to double-blind studies, often acquire data on the incidence rate of side effects and the effects of a new drug to track the impact of prolonged therapy. Regardless, open-label practices have several drawbacks. In open-label studies, there may be ethical problems as well as ascertainment bias, which can affect the objectivity of the study. To obtain more accurate results, a study should involve a double-blind or even triple-blind study.

##### 3.6.3.4 Clinical obstacle information

As DRM barriers can be found through recent bursts, we believe that “accumulation,” “sex,” and “pretreatment drug resistance (PDR)” have been hindrances and troublesome in recent years. Accumulation of deleterious mutations is caused by elevated turnover of lymphocytes (Galvani, 2005). This may result in fewer therapeutic alternatives available, the possibility of DRM spread, and the acceleration of the immune system’s aging process (Galvani, 2005; Villabona-Arenas et al., 2016). Since accumulation is nonlinear, selection is restricted to genetic variation (Maldarelli et al., 2013), and additional investigation is required to ascertain the causes of such selection. Pretreatment mutation testing must be performed in locations where antiretroviral therapy (ART) is used for an extended period, and more research into the process and conditions for mutation accumulation is needed.

The commonly used NNRTIs efavirenz and nevirapine are typically the cause of pretreatment drug resistance (PDR) (Ndashimye and Arts, 2019), which is frequently followed by acquired drug resistance and greater risk of virologic failure (Parikh and Mellors, 2012). Although PDR is uncommon, it has increased in the last several years (Kiros et al., 2023). Transmission of the related mutations K103NS and E138A still occurs (García-Morales et al., 2021). Notably, high-incidence patient groups of PDR include men who have sex with men (MSM), sex workers, and inmates (Macdonald et al., 2020). MSM are especially susceptible to HIV transmission through sexual activity, as HIV can be spread through rectal anal contact without the use of a condom. Bacterial sexually transmitted infections may also be a contributing factor by facilitating HIV transmission (Macdonald et al., 2020). As a result, these groups need to receive more attention, and appropriate coping strategies must be outlined to address the rising PDR.

## 4 Discussion

Increasing emphasis is being placed on DRMs since antiretroviral medications are essential for first-line clinical treatment. Over the past decade, research on DRMs has advanced rapidly. However, much remains to be learned about the mechanism of mutation, data on cases of resistant, and development of innovative therapeutics. Analyzing the past, present, and future development and research directions is essential for improving and innovating therapeutic outcomes. Based on 3,694 documents created between January 1, 2011, and December 31, 2023, this research presents scientometric and visual assessments of DRMs and visualized analyses of the hot research topics and frontiers in this area.

### 4.1 General information

In 1991, the initial paper on DRMs in WoSCC examined ganciclovir mutations in advanced HIV patients with CMV infection (Drew, 1991). Following this study, further drug-related tests were carried out to obtain additional information or to determine the mutation mechanism involved. The number of papers in this field has shifted in recent years. The growing use of NGS has spurred many attempts to identify mutation mechanisms of HIV. Another potential cause of this is related to virus mutations and the unpredictable nature of transmission. The United States accounts for the majority of papers on DRM research. The 5 counties with the most publications are represented by 2,851 (77.1%) papers in this field. England has the highest centrality among them, suggesting that it has been a crucial hub for international integration and transfer of HIV DRM data. In addition, the top five institutions in terms of publications have contributed 899 (24.3%) papers in this field. These results indicate highly collaborative multinational research. However, scholars need to focus on low-resource areas to obtain more comprehensive global statistical results. The top 10 prolific authors contributed a total of 452 papers. Nonetheless, there are 8 authors whose centrality is less than 0.1. This is not a particularly high quantity, which suggests that further collaboration between these productive authors is necessary. Among them, a report on INSTI resistance was conducted by Wainberg, M. A., and Anstett, K. The review covered all available information on five distinct kinds of strand-transfer integrase inhibitors, including the resistance state of inhibitors to various viral subtypes (Anstett et al., 2017); the review assisted in integrating the current state of INSTI mutations and directed the creation of novel medications. The 3 most-cited works in the literature are those by Bennett et al. (2009), Johnson et al. (2011), and Gupta et al. (2018). Their efforts have unified the genetic definition, first-line treatment, and statistical analysis of the worldwide HIV infection status. Based on the results of the overlay map, we found that, except for immunology and clinical medicine, DRMs are associated with numerous sociologically relevant professions, which shows that this subject has a strong influence in various fields.

By analysis of keywords, the terms “binding pocket,” “tolerant region,” “molecular dynamics,” and “reduce response” are among the keywords that compose the most extraordinary cluster of this term. In-depth studies on the aforementioned topics can direct the creation of novel medications and elucidated the processes underlying viral alterations. Other clusters indicate that low-level viremia (LLV) and molecular dynamics (MD) are the main research areas in DRMs. Although nations have varied definitions of LLV, it is generally accepted that patients receiving ART have persistent virus loads between 50 and 1,000 copies/mL (Bai et al., 2022). According to a report on resistance mutations in HIV-1 patients following first-line therapy, the rate of mutations in this population is high: 37% of patients had newly discovered resistance mutations, with M184I/V, K103N, and M230L being the most common (Taiwo et al., 2011). In light of the above findings, testing for resistance mutations is required to prevent medical invalidation, and it is recommended that patients with LLV undergo high-sensitivity testing regularly to prevent a potential chronic risk of disease. MD simulations have been extensively employed in research on the stability and dynamic properties of proteins and protein–ligand complexes. MD simulations can be used to investigate the molecular mechanism of resistance (Rana et al., 2022). Since commonly utilized MD simulations favorably influence the resistance mechanism, we propose that a mechanism-related research force use this technique. Regarding the timeline, the best time frame for DRMs was between 2010 and 2015. During this time, several terms related to DRMs were found, such as cohort, adherence, *Mycobacterium tuberculosis*, and cleavage site. Adherence is a noteworthy keyword. There is already evidence to suggest that high adherence contributes to reducing the occurrence of mutations through complete suppression of viral replication (Lucas, 2005). This was a positive development for blocking the virus from spreading and decreasing the likelihood of DRMs. Patients should use drugs with low resistance potential and closely adhere to dosage instructions.

### 4.2 Emerging topics

Based on an analysis of the results of citation bursts, “next-generation sequencing,” “tenofovir alafenamide,” “children,” “accumulation,” “dolutegravir,” “rilpivirine,” “sex,” “pretreatment drug resistance,” and “open label” are the boundaries for this subject. For first-line therapy regions, there are two terms highlighted in our results: DTG and NGS. First-line treatment is recommended to consist of combination medicine therapy based on DTG because it has fewer side effects than other reported antiretroviral drugs. Therefore, newer antiretroviral drugs should be developed for such patients. Moreover, widespread use of NGS in the field of resistance mechanisms has led to an update in sequencing methods and emergence of more sophisticated sequencing techniques relying on NGS. This phenomenon aids in the search for the mechanism underlying mutation of HIV. In addition, since 2020, open-label research has gained much attention; it is typically used to determine tolerability and safety. This indicates that more novel medications have entered clinical trials, meaning that patients will have a wider selection of drugs. According to recent reports, the rate of new pediatric infections has remained high (Ka'e et al., 2023). NNRTI-based regimens are the initial choice for these populations (Agwu et al., 2014). Fortunately, there is less drug pressure due to restricted drug selection, which lowers the rate of mutation in this population (Agwu et al., 2014). TAF is a novel medication that was recently released, and further clinical trials are needed to identify any side effects. Furthermore, medical professionals need to pay closer attention to RPV-associated mutations such as K103N and M184I/V, and relevant drug resistance management techniques need to be developed.

### 4.3 Obstructions and prospects

In general, even with ongoing approval of new medications, the state of HIV resistance is still dire, and different levels of drug resistance have an impact on clinical treatment. Although the availability of DTG has improved outcomes, additional research is required to identify any potential adverse effects. Binding sites and current medications need to be further developed to support future studies on novel treatments. The development of NGS has significantly decreased the sequencing time and maintained the high accuracy of virus mutation detection in investigations of drug resistance mechanisms. However, additional technologies can still be integrated with it to obtain even greater efficiency. The challenges in treating AIDS are the increasing prevalence of PDR and accumulation of drug-resistant mutant genes. Furthermore, a significant obstacle to HIV treatment is the ability of the previral DNA reservoir to store gene alterations. Therefore, there must be more effort in future research to address these two issues, such as finding better drug combinations for patients with PDR and multidrug resistance mutations and routine monitoring of patients receiving long-term ART. For clinical practice, we propose that open-label studies, which are frequently carried out, should be undertaken through double-blind testing to increase the objectivity of results. Although studies on teenagers have increased recently, there are still many unclear facts about this age group, and their ability to use certain drugs is also restricted. Patient adherence is a major factor in the impact of clinical treatment. Therefore, selecting medications with a longer half-life and administering them once a day can help with patient compliance.

## 5 Limitations

Limitations, which results in inadequate data collection. Additional research on papers from other databases is needed. Furthermore, as time progresses, more articles will be published. Hence, the results of the analysis might not match the actual circumstances.

## 6 Conclusion

This study reports the number of publications, cooperation of authors and nations, journals, references, and keywords, and a visualization analysis of publications on drug resistance mutations. As a result of CiteSpace’s visualization analysis, the majority of conclusions are from low- and middle-income countries. Considerable progress has been made in drug mutation, mutation mechanism, and HIV mutation detection techniques. The frontiers in this field included DTG, pediatric patients, NGS, open-label sequencing, *M. tuberculosis*, and others. Subsequent research should concentrate on medication resistance in teenagers, addressing pretreatment drug resistance-related concerns and exploring the adverse effects of recently published medications, such as TAF.

## Data availability statement

The original contributions presented in the study are included in the article/Supplementary material, further inquiries can be directed to the corresponding author.

## Author contributions

XNC: Validation, Writing – original draft, Writing – review & editing. XC: Validation, Writing – review & editing. YL: Conceptualization, Funding acquisition, Supervision, Writing – review & editing.
